# Lipid-lowering fibrates extend *C. elegans* lifespan in a NHR-49/PPARalpha-dependent manner

**DOI:** 10.18632/aging.100548

**Published:** 2013-04-08

**Authors:** Sven Brandstädt, Kathrin Schmeisser, Kim Zarse, Michael Ristow

**Affiliations:** ^1^ Department of Human Nutrition, Institute of Nutrition, University of Jena, Jena D-07743, Germany; ^2^ Energy Metabolism Laboratory, Swiss Federal Institute of Technology (ETH) Zurich, Schwerzenbach/Zürich, CH-8603, Switzerland; ^3^ Department of Clinical Nutrition, German Institute of Human Nutrition Potsdam-Rehbrücke, Nuthetal D-14558, Germany

**Keywords:** ageing, lifespan, healthspan, C. elegans, nematodes, pharmacology, nuclear receptors, peroxisomes, peroxisome proliferator activated receptor alpha, PPARalpha, beta oxidation

## Abstract

Compounds that delay aging in model organisms may be of significant interest to anti-aging medicine, since these substances potentially provide pharmaceutical approaches to promote healthy lifespan in humans. We here aimed to test whether pharmaceutical concentrations of three fibrates, pharmacologically established serum lipid-lowering drugs and ligands of the nuclear receptor PPARalpha in mammals, are capable of extending lifespan in a nematodal model organism for aging processes, the roundworm *Caenorhabditis elegans.*

Adult *C. elegans* (wild-type N2 as well as two *nhr-49*-deficient strains, RB1716 and VC870) were maintained on agar plates and were fed *E. coli* strain OP50 bacteria. Bezafibrate, clofibrate, and fenofibrate were applied to the agar, respectively, to test whether they may promote longevity by quantifying survival in the presence and absence of the respective compounds.

All three fibrates extended *C. elegans* N2 lifespan when applied at a concentration of 10 micromolar. Bezafibrate additionally extended *C. elegans* N2 lifespan at concentrations of 1 micromolar and 0.1 micromolar. In strains deficient for *nhr-49*, a functional orthologue of the mammalian peroxisome proliferator-activated receptor alpha (PPARalpha), all three compounds were incapable of extending lifespan.

Taken together, fibrates promote *C. elegans* longevity in an NHR-49-dependent manner possibly by promoting mitohormesis and suggesting that these compounds may promote lifespan also in mammals.

## INTRODUCTION

Promotion of longevity and in particular extension of healthy lifespan (also named ‘healthspan’) is of eminent interest to most humans. Specific mutations have been shown to extend the lifespan of model organisms dramatically [1-8], while more readily available interventions, including calorie restriction, also extend life expectancy of model organisms [9, 10].

Accordingly, considerable effort has been invested to identify naturally occurring and/or pharmaceutical compounds that promote longevity in model organisms. A number of such compounds have been identified in recent years, including rapamycin [11-15], resveratrol [13, 16-19], 2-deoxy-D-glucose [20], lithium [21, 22], glaucarubinone [23], lonidamine [24], rotenone [25], and others reviewed elsewhere.

Fibrates are amphipatic carboxylic acids that are used to treat metabolic disorders, primarily hypercholesterolemia and/or hypertriglyceridemia [26]. As lipid-modifying substances they are capable of increasing HDL cholesterol levels and decreasing triglycerides and LDL in plasma [26]. Like certain fatty acids, they are agonists of the peroxisome proliferator activated receptors (PPARs). Activation of PPAR by fibrates leads to increased hydrolysis of triglycerides, stimulation of cellular fatty acid uptake and conversion to acyl-CoA derivatives, decreased synthesis of triglycerides and fatty acids as well as VLDL, and finally increased peroxisomal and mitochondrial beta oxidation [26].

PPARs are nuclear receptors that act as transcription factors. They typically heterodimerize with the retinoic X receptor (RXR) and regulate expression of genes involved in development, metabolism, and cellular differentiation after binding to their respective response elements (PPREs) [27]. PPARs regulate expression of genes involved in intra- and extracellular lipid metabolism, especially genes implicated in beta oxidation [28]. PPARalpha is a master regulator of lipid metabolism. Activation induces the expression of the liver-X-receptor and ACBA1, a transporter that mediates cholesterol efflux from macrophages [29]. Furthermore, it controls adaptive response processes to calorie restriction due to its ability to activate ketogenesis [30].

The nematodal nuclear hormone receptor 49 (NHR-49) has sequence homology to the human hepatocyte nuclear factor 4 (HNF4), but is assumed to act as the functional orthologue of mammalian PPARalpha since it shares most of the biological activities of the latter [31].

In this study we have tested whether various fibrates, namely bezafibrate, clofibrate, and fenofibrate, at pharmaceutical doses may be capable of extending the life span of the nematodal model organism *C. elegans*.

## RESULTS

### Fibrates extend *C. elegans* life span

By continuously exposing nematodes starting at young adult age for their entire lifespan to defined concentrations of three different fibrates, which in mammals serve as ligands for the nuclear receptor PPARalpha, we tested whether and to which extent these compounds affect *C. elegans* lifespan.

**Figure 1:**
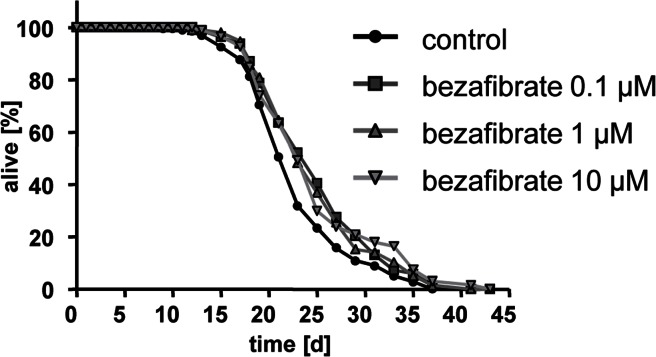
Bezafibrate extends lifespan of adult *C. elegans* Life span analyses with several hundred N2 wild type nematodes each at three different concentrations of bezafibrate (0.1, 1, and 10 micromolar) versus control (0.1% DMSO).

Bezafibrate extended nematodal life span at three different concentrations (0.1, 1, and 10 micromolar) (Fig. 1). The maximum observable effect on mean life span was 2.8 days which occurred at a concentration of 10 micromolar (pls. see Table 1 for details, also applies to all following life span assays).

**Table 1:** Results and statistical analyses of life span assays

Strain	Treatment	Max Life Span in Days ± SD	Mean Life Span in Days ± SD	n	p-Value versus Control	
N2	0.1 % DMSO control	28.1 ± 1.5	21.6 ± 1.2	11		
	clofibrate 0.1 μM	25.9	22.0	1	n. s.	0.8248
	clofibrate 1 μM	27.2	22.6	1	n. s.	0.1877
	clofibrate 10 μM	33.8	25.1	1	***	< 0.0001
	clofibrate 10 μM	34.2 ± 0.9	23.0 ± 0.5	3	***	< 0.0001
	bezafibrate 0.1 μM	32.0	23.3	1	*	0.0138
	bezafibrate 1 μM	33.1	22.8	1	*	0.0137
	bezafibrate 10 μM	34.4	22.9	1	**	0.0078
	bezafibrate 10 μM	32.5 ± 1.8	24.4 ± 0.7	3	***	0.0002
	fenofibrate 0.1 μM	34.6	22.5	1	*	0.0143
	fenofibrate 1 μM	28.8	22.3	1	n. s.	0.1824
	fenofibrate 10 μM	32.8	25.6	1	***	< 0.0001
	fenofibrate 10 μM	30.2 ± 0.3	23.7 ± 1.1	3	*	0.0218
						
*nhr-49*						
ok2165	0.1 % DMSO control	17.2 ± 1.2	12.2 ± 0.4	3		
	bezafibrate 10 μM	17.7 ± 0.8	11.5 ± 0.2	3	n. s.	0.1606
	clofibrate 10 μM	15.7 ± 0.4	11.0 ± 0.2	3	neg. s.	< 0.0001
	fenofibrate 10 μM	13.5 ± 0.2	9.5 ± 0.0	3	neg. s.	< 0.0001
						
gk405	0.1 % DMSO control	16.7 ± 3.6	11.0 ± 0.4	3		
	bezafibrate 10 μM	17.7 ± 3.7	11.4 ± 0.8	3	n. s.	0.0679
	clofibrate 10 μM	17.5 ± 3.4	11.0 ± 0.3	3	n. s.	0.8962
	fenofibrate 10 μM	14.5 ± 1.6	10.8 ± 0.2	3	n. s.	0.3035

**Figure 2:**
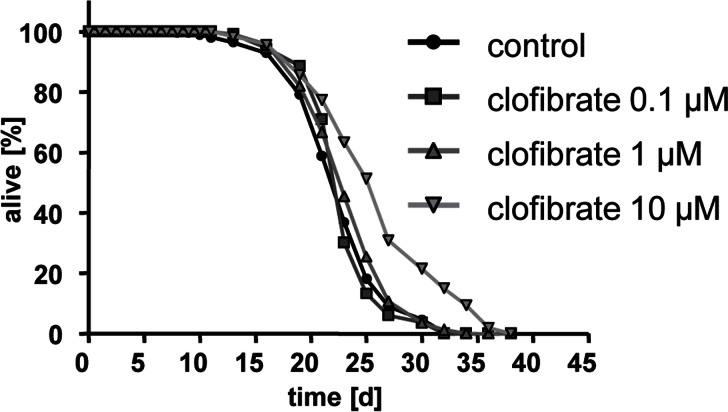
Clofibrate extends lifespan of adult *C. elegans* Life span analyses with several hundred N2 wild type nematodes each at three different concentrations of clofibrate (0.1, 1, and 10 micromolar) versus control (0.1% DMSO).

Clofibrate cause extension of *C. elegans* lifespan at a concentration of 10 micromolar (Fig. 2) reflected by a mean life span of 23.0 days equaling an increase of 1.4 days.

**Figure 3:**
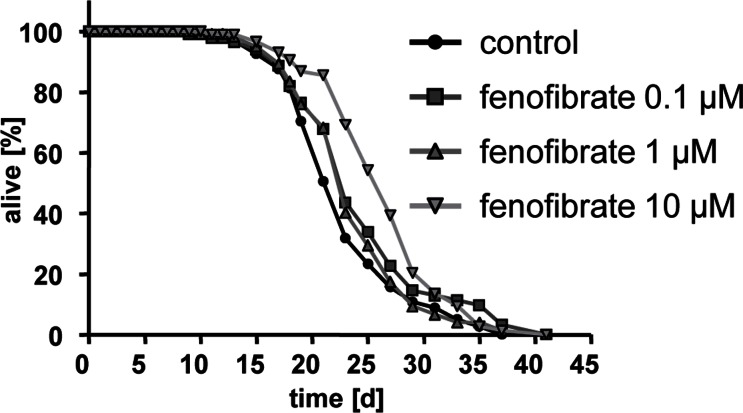
Fenofibrate extends lifespan of adult *C. elegans* Life span analyses with several hundred N2 wild type nematodes each at three different concentrations of fenofibrate (0.1, 1, and 10 micromolar) versus control (0.1% DMSO).

Fenofibrate was capable of promoting life expectancy at concentrations of 0.1 and 10 micomolar (Fig. 3) with the most pronounced increase in mean life span at a concentration of 10 micromolar as reflected by an increase of 2.1 days in comparison to wild-type worms.

The effects on maximum life span (80^th^ percentile) were an extension by 4.4 days for bezafibrate (10 micomolar), an extension by 6.1 days for clofibrate (10 micromolar), and an extension by 6.5 days for fenofibrate (0.1 micromolar).

Taken together, these findings indicate that three different fibrates are capable of extending both mean and maximum lifespan of wild type *C. elegans.*

### Life span extension through fibrates is PPA-Ralpha/NHR-49 dependent

As stated in the introductory section fibrates serve as well-accepted ligands for the mammalian PPARalpha, a nuclear receptor known to heterodimerize with the retinoid-X-receptor (RXR) to promote a number of catabolic processes. The nematodal nuclear hormone receptor 49 (NHR-49) is commonly accepted to serve as a functional orthologue of mammalian PPARalpha since it shares most of the biological activities of the latter [31].

**Figure 4:**
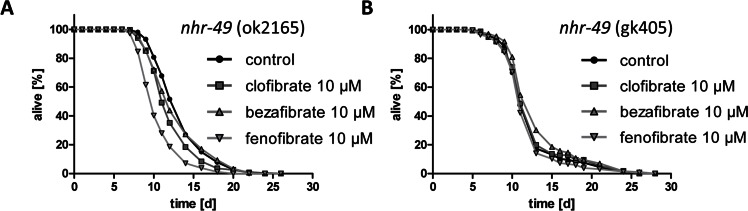
Life span extension through fibrates is NHR-49/PPARalpha-dependent **A** Life span analyses with several hundred *nhr-49* knockout nematodes (variation ok2165, strain RB1716) at 10 micromolar bezafibrate, clofibrate, and fenofibrate versus control (0.1% DMSO). **B** Life span analyses with several hundred *nhr-49* knockout nematodes (variation gk405, strain VC870) at 10 micromolar bezafribrate, clofibrate, and fenofibrate versus control (0.1% DMSO).

To test the hypothesis whether fibrates act as agonists of PPARalpha and therefore extent life span in *C. elegans*, the life span assays were repeated using two strains that lack functional *nhr-49*, variation ok2165 and variation gk405 (strains RB1716 and VC870, respectively). For this, the most effective life span extending fibrates concentration in wild type, 10 micromolar, was used. Consistent with the hypothesis, clofibrate, bezafibrate, and fenofibrate failed to extend nematodal life span in absence of NHR-49 (Fig. 4A and 4B).

## DISCUSSION

To potentially support the ongoing search for compounds that may promote human health especially at higher age, we here show that the fibrates clofibrate, bezafibrate, and fenofibrate induce longevity in a nematodal model organism, the roundworm *C. elegans*. Interestingly, these effects are dependent on the nematodal orthologue of PPARalpha, NHR-49, mediating the promotion of life span.

The *C. elegans* PPARalpha orthologue NHR-49 induces the expression of genes involved in energy metabolism, more precisely in fatty acid beta oxidation (*acs-2*, *ech-1*), desaturation (*fat-5*, *fat-7*), transport, and synthesis of mono-methyl branched-chain fatty acids [31, 32]. It remains to be elevated, which of these mechanism are responsible for the life span extending effect of fibrates. Due to stimulation of fatty acid beta oxidation an increase in ROS formation may occur [33], which could promote formation of reactive oxygen species (ROS) in nematodes [20, 23]. This increase in ROS may act as a signal to increase stress response and antioxidant defense resulting in longevity resembling an adaptive response signaling process that was named mitochondrial hormesis or mitohormesis [20, 34, 35]. Based on the current findings, it appears feasible that fibrates act by employing a similar mechanism, especially since it is shown that PPARalpha agonists increase the expression of superoxide dismutase, a major enzyme in antioxidative defense [36].

It is unclear whether our results can be extrapolated to mammals or even humans since the current study has been performed in the model organism *C. elegans*. However, other compounds that have been identified by using a similar, metazoan-based approach have been shown to be effective also in rodents [11-19].

Summarizing these findings, it is likely that fibrates acting as agonists of PPARalpha to promote health and life span through modulating beta oxidation and ROS formation in a mitohormetic manner, suggesting that these substances may be potential to prevent aging and age-associated diseases also in higher organisms.

## METHODS

### Compounds

Bezafibrate, clofibrate and fenofibrate were obtained from Sigma-Aldrich (Munich, Germany).

### *C. elegans* maintenance

The *C. elegans* strains used were Bristol N2, as well as the mutant strains *nhr-49(ok2165)* and *nhr-49(gk405)*. These were obtained from CGC. Maintenance was performed as previously described [24]. The *E. coli* OP50 strain was used as food source.

### Life span assays

Compounds were admitted to the agar as previously described [24]. *E.coli* OP50 bacteria were heat-inactivated for 45 minutes as previously described to avoid interference by the xenobiotic-metabolizing activity of E. coli, and used as the only food source [37].
